# Navigating new pathways – healthcare personnel’s experiences transitioning from traditional nursing homes to the dementia village care model in Norway

**DOI:** 10.1186/s12913-026-14223-9

**Published:** 2026-02-20

**Authors:** Daniela Lillekroken, Eva Elisabeth Hessevaagbakke, Ellen Karine Grov, Lene Strandberg Hansen, Olav Johannes Hovland, Anne-Marthe Rustad Indregard, Katrin Lindeflaten, Kari Röhrl, André Strømstad, Astrid Cathrine Vik Torbjørnsen, Anne-Martha Utne Øygarden, Christine Tørris, Ann Kristin Bjørnnes

**Affiliations:** 1 Department of Nursing and Health Promotion, Faculty of Health Sciences, Oslo Metropolitan University, PB4 St. Olavs Plass, Oslo, N-0130 Norway; 2 Furuset Hageby, Granstangen 51, Oslo, 1051 Norway; 3 Dronning Ingrids Hage, Hovinveien 6, Oslo, 0576 Norway; 4 Department of Nursing and Health Sciences, Faculty of Health and Social Sciences, University of South-Eastern Norway, Campus Vestfold, Raveien 215, Borre, 3184 Norway

**Keywords:** Dementia, Dementia villages, Focus groups, Healthcare personnel, Nursing homes, Person-centred care, Qualitative content analysis

## Abstract

**Background:**

The global rise in dementia has led to growing interest in care models that promote autonomy, dignity, and well-being for people living with dementia. In response, dementia villages, pioneered in the Netherlands, have emerged as innovative alternatives to traditional nursing homes, integrating daily life routines with secure, dementia-friendly environments. Norway has followed and established dementia villages in four municipalities. Despite the innovative appeal of dementia villages, little is known about their impact on healthcare personnel’s care activities. This study aims to explore healthcare personnel’s experiences transitioning from traditional nursing homes to the dementia village care model in Norway.

**Methods:**

The study employs an explorative-descriptive research design. Ten focus group interviews, involving a total of 53 healthcare personnel employed at two dementia villages, were conducted between October and November 2024. The data were analysed using qualitative inductive content analysis. The study is reported following the Consolidated Criteria for Reporting Qualitative Research (COREQ).

**Results:**

The data analysis revealed that healthcare personnel viewed their experiences working in dementia villages as opportunities for professional growth, innovation, and adaptation to a new care environment. This perspective is captured in three overarching categories: (i) Adaptation in a new care environment; (ii) Managing residents’ increased freedom of movement; and (iii) Development of the caregiver role within DVs. Together, these categories illustrate a shift away from traditional models of dementia care toward more flexible and person-centred approaches, in which healthcare personnel, despite challenges, adapt their roles and practices to meet residents’ individual needs through innovative and creative caregiving solutions.

**Conclusion:**

This study provides a nuanced understanding of how healthcare personnel experience their evolving roles within dementia villages. While the dementia village model supports resident autonomy, healthcare personnel reported several challenges in balancing the opportunities of person-centred care within a new physical environment. To fully realise the potential of dementia villages, caregiving roles must be supported through tailored training, appropriate staffing, and opportunities for professional growth that preserve staff identity and competence.

**Supplementary Information:**

The online version contains supplementary material available at 10.1186/s12913-026-14223-9.

## Background

The ageing population is one of the significant demographic changes many countries face in the twenty-first century [1], including Norway [2]. Older age is also characterised by the emergence of several complex health states commonly called geriatric syndromes, one of them being dementia [3]. Globally, more than 50 million people are living with Alzheimer’s or other forms of dementia, a number that will triple by 2050 [3]. National governments have increasingly drawn attention to the challenge of supporting people with dementia to live longer in their community; therefore, the 2013 G8 dementia summit called for governments to “disseminate successful approaches to supporting people with dementia and their caregivers, including … community-based programs fostering inclusion and improved quality of life” [3, 4]. Creating age-friendly environments will enable older people to age safely in a place that is right for them, continue to develop personally, be included, and contribute to their community while retaining their independence and health [4]. To meet the needs for healthcare services, the Norwegian government proposed and implemented the Dementia Plan 2025 [5], an action plan designed to contribute to a more dementia-friendly society.

In Norway, there are more than 101,000 persons living with dementia, and over 84% of those with long-term placements in nursing homes have cognitive impairment or a form of dementia [6, 7]. Results from a National Survey indicate that in 2022, 1,577 special care units with 12,551 beds were allocated for people with dementia [8]. Since the early 1980s, there has been a trend toward developing small, well-adapted special care units for people with dementia living in nursing homes [9]. From the mid-1990s, there have been significant developments in policy, with residential provision shifting towards the establishment of special care units for people with dementia [5]. Moreover, since the late 1990s, social models have also been introduced into dementia care, emphasising an understanding of the emotions and behaviours of people with dementia within the framework of their social context and personal history [10].

Currently, there is no pharmacological cure for dementia [3]; therefore, a range of changes in the philosophy of care were gradually introduced within dementia care. In sharp contrast to traditional models of care, which refer to conventional dementia care in traditional nursing homes with institutional routines and environments [11], the concepts of ‘culture change’ [12] and ‘person-centred care’ [13] describe changes in the philosophy of care, the architecture, and the organisational patterns of traditional institutions [14]. In Norway, traditional nursing homes are municipal, publicly funded long-term care facilities that provide 24-hour health and personal care to older adults who can no longer live independently due to complex health or functional needs [15]. Over 80% of the residents have dementia and may experience neuropsychiatric symptoms such as agitation, depression, and anxiety [16]. Staffing typically includes registered nurses, nurse aids, and care assistants, with ongoing challenges related to workforce qualifications, recruitment, and workload [17]. Although intended to deliver both medical, rehabilitative, and daily care, these nursing homes have been criticised for routine-driven institutional care that feels less homelike and limits autonomy [15, 18]. The physical environment is typically institutional, with interior and exterior spaces designed primarily to meet residents’ fundamental care needs rather than to promote homelike living or resident autonomy. Such facilities often consist of long corridors, shared common areas, and private or semi-private rooms, which are furnished primarily for functionality rather than personal comfort. The design still resembles “total institutions” where routines dictate daily life, and individual living spaces may retain hospital-like qualities, an environment that constrains opportunities for meaningful activities, outdoor access, garden and village opportunities, and engagement with nature, factors that residents often identify as crucial for well-being and thriving [19].

Overall, the physical environment in many traditional nursing homes may not fully support person-centred care or homelike living, and this limitation is increasingly addressed in quality improvement and architectural discussions within Norwegian long-term care [19]; therefore, a culture change has been welcomed as a transformation rooted in values and beliefs that empower residents in care settings [20]. The ultimate vision is through a cultural change to create a culture of aging that is life-affirming, satisfying, humane, and meaningful, seeking to transform a ‘facility’ into a ‘home’, a ‘resident’ into a ‘person, and a ‘schedule’ into a ‘choice’ [20]. Culture change relies heavily on the incorporation of person-centred care, which refers to the practice of basing key decisions on a resident’s needs, desires, and preferences in areas ranging from how meals are served and how bathing is offered to how work is structured within an organisation [21–23]. Within the culture of change movement, significant systematic changes occur at all levels, including the physical environment, mealtimes, relationships, and the flattening of the traditional staff hierarchy, which creates more opportunities for caregivers to get to know the residents and understand their needs, thereby meeting them more effectively [24].

The way individuals perceive their environment significantly affects their ageing process; an unhealthy environment can have detrimental effects, increasing frailty and vulnerability [25]. Therefore, over the last decades, there has been a trend to improve the quality of nursing home environments through the deinstitutionalisation of these facilities by creating dementia-friendly environments and incorporating outdoor natural landscapes, such as sensory gardens [26, 27]. An outdoor garden is a significant environmental initiative that can provide comfort, foster connections to leisure interests, encourage social interactions and activities, and promote a sense of agency by offering individuals the opportunity to step away from the healthcare setting temporarily [28]. The small house model is an attempt to deinstitutionalise care, aiming to improve the physical environment and provide a safe, familiar environment for a person living with cognitive impairment [29]. When a person with dementia experiences comfort, attachment, inclusion, and agency, they are likely to exhibit higher levels of intersubjectivity and improved overall well-being [13, 30, 31].

The need for people to embrace nature has led to the establishment of a pioneering care facility, the Dutch Hogewey nursing home, which opened in 2009 and was the world’s first and, at that time, the only village for residents living with dementia. This dementia village^1^ [DV] was touted as a place where people could live and die in a communal setting, stripped of the impersonal hospital feel and clinical smell that most nursing homes still exude [32]. Since 2009, more than ten DVs have been established worldwide, with seven in Europe and three in Canada, New Zealand, and Australia. The DV model supports autonomy, unique needs, and the continuity of daily living patterns through housing integrated with extensive exterior walks and gardens, as well as access to restaurants, grocery stores, pubs, and a theatre, all within familiar, everyday surroundings, thereby reducing residents’ anxiety and fear [33]. Although this pioneering health care model negotiates rivalling discourses of intimacy, professionalisation, and medicalisation [32], it demonstrates that people can live in a secure setting while continuing to receive the daily stimulation from the things they love to do, like exercising outside and attending classes and clubs, with simultaneous access to medical attention and care services they may need [34].

Following the successful launch of the first DV in the Netherlands, the Norwegian government chose to develop similar village-style communities to promote mobility and an active daily life for people with dementia [35]. This initiative has resulted in the construction of four DVs across three Norwegian municipalities: two in Oslo, one in Bærum, and one in Kristiansand.

Despite DVs’ innovative appeal and increasing adoption worldwide, results from a recently conducted scoping review [36] indicate that the scientific literature on the DV model of care remains limited. However, in recent years, a small number of scholars have begun to address this gap by conducting exploratory and evaluative studies to examine the implementation, outcomes, and potential benefits of the DV model for people living with dementia. The impact of opening a DV within a French community on the general population was recently examined by Pech et al. [37]. The researchers surveyed residents of Dax, France, before and after the opening of DV in 2020. The survey’s results indicate that, following the village’s opening, attitudes toward Alzheimer’s disease among the general population became more positive. The DV was explicitly designed to enable older adults with Alzheimer’s disease to live a “normal life” in an environment that is open to the city and accessible to the general public, thereby conveying a markedly different image from that traditionally associated with nursing homes.

A Danish study [38] explored how relatives of people with dementia and healthcare professionals work to create and maintain a meaningful everyday life for the residents in DV. Results from the study demonstrate that both relatives and healthcare professionals were committed to creating a meaningful everyday life for the residents, but also reveal different understandings of when, where, and how this could be achieved. Furthermore, people with advanced dementia may not be able to benefit from the activities and possibilities provided by the DV, since this requires resources beyond what can be provided.

While most research regarding DVs has focused on the architecture and design of the village [39], no study has yet been conducted to investigate whether DVs improve behaviour, functional ability, cognition, and quality of life for residents [36]. Moreover, to the best of our knowledge, no study has examined healthcare personnel’s experiences of transitioning from traditional nursing homes to the dementia village care model in Norway. Although earlier studies explored how the physical environment in traditional nursing homes affects healthcare personnel’s experiences in dementia care [40, 41], no study has used DVs as a research context. Gaining clearer insights into healthcare personnel’s experiences will be beneficial for evaluating this care model and assessing its viability in dementia care; therefore, further research is needed.

## Methods

### Aim and design

This study aims to explore healthcare personnel’s experiences transitioning from traditional nursing homes to the dementia village care model in Norway; therefore, the research question is: “What are healthcare personnel’s experiences transitioning from traditional nursing homes to the dementia village care model in Norway”? The study has an exploratory-descriptive qualitative (EDQ) research design [42] based on focus group interviews with healthcare personnel employed at two Norwegian DVs. This approach emphasises understanding participants’ experiences and perceptions, providing rich, detailed descriptions that better reflect real-world contexts. Furthermore, this approach is advantageous when there is limited prior research on a topic (in this case, DV as a care model in dementia care), as it provides a structured yet open-ended way to uncover new insights and deepen understanding. The Consolidated Criteria for Reporting Qualitative Research (COREQ) [43] were followed in the reporting of the study (Supplementary File).

### Study setting

The study was conducted in two Norwegian DVs. The first DV (DVA) can accommodate up to 130 residents in units of 6–8 residents, with a few units accommodating up to 10 residents. At the time the interviews were conducted, in units with 10 residents, the healthcare personnel-resident ratio was 1:5. In contrast, in the smaller units, the ratio was 1:6. The second DV (DVB) has a capacity of 112 places, divided into 14 small living units, each accommodating eight residents, with 32 places reserved for younger people with dementia. All residents in both DVs have their private rooms, each equipped with a bathroom. Each room featured large windows and modern furniture. The interior design has walls painted in light, bright colours. The doors to residents’ rooms are painted in contrasting colours to facilitate easy room identification and ensure a homely environment. The residents have direct access to the outdoor area from a common area. Both DVs are municipally owned and operated. The extensive use of care technology, such as electronic patient journals, location-tracking technology-enabled wristbands, digital monitoring systems, and DVs’ communication platforms, supports residents’ safety. The surrounding gardens are fully fenced, ensuring a secure environment. Each DV has sensory gardens, with various art objects placed for easy viewing. The DVs also feature a restaurant, café, shop, and cultural hall available for a range of activities, bringing a touch of city life into the village for the enjoyment of residents, their family members, and other visitors. Both DVs are staffed with healthcare personnel 24 h a day, indicating a need for advanced healthcare among the residents. The healthcare personnel’s workday starts with a quiet report, followed by planning for the day. The initial responsibilities included assisting residents with personal hygiene and preparing meals, which may also involve grocery shopping if needed. Nurses, in addition to their other healthcare and administrative duties, are responsible for distributing medication to residents. Between meals, healthcare personnel perform various cleaning and maintenance tasks. These include tidying residents’ rooms, keeping the kitchen and bathrooms clean and ready for use, doing laundry, and changing bed linens. Even with limited time to manage responsibilities, healthcare personnel also prioritise spending quality time with residents and walking in the gardens, regardless of the weather.

### Participants’ recruitment

To recruit participants, the researchers first submitted a formal application to the DV’s management outlining the study’s aims, methodology, and ethical considerations. Upon approval, some of the researchers met with the quality managers at each DV, explained the study’s purpose in detail, and requested their assistance with participant recruitment. Recruitment was supported through quality managers’ verbal announcements describing the study’s focus, eligibility criteria, and voluntary nature. An informational meeting was subsequently held, during which some of the researchers met with the quality managers, other members of the leadership, and some staff members, provided a comprehensive explanation of the study, and addressed any questions or concerns. Following this meeting, the researchers and the quality managers agreed on a timeframe for conducting the focus group interviews.

A purposive sampling strategy was used to recruit healthcare personnel, involving the identification and selection of individuals with specific characteristics who were well-informed and proficient, and who would be better able to assist with the research [44]. Therefore, potential candidates were recruited based on the following inclusion criteria: being healthcare personnel with experience in dementia care, employed at one of the DVs, and having sufficient proficiency in Norwegian to understand and express themselves. These criteria ensured that participants possessed both contextual knowledge of the DVs’ setting and professional experience relevant to the provision of care for people living with dementia. The period between when healthcare personnel transitioned to a DV and when the interviews were conducted varied: one and a half years for the DVA and nine months for the DVB.

The researchers did not know the participants before the data collection. Although some authors have clinical experience in dementia care, none of the researchers involved in this study had a prior professional relationship with the DVs or the participants, and this positionality was made explicit during recruitment to minimise perceived coercion. The quality managers were involved only in participant recruitment, and none of the researchers in the current study held managerial roles with respect to participants, thereby reducing potential power imbalances and reducing participants’ sense of obligation to participate.

### The participants’ characteristics

A total of 53 healthcare personnel, comprising 27 participants from DVA and 26 from DVB, participated in 10 focus group interviews. The focus groups comprised healthcare personnel scheduled to work on the day of the sessions. To ensure maximum variation in sampling by age and work experience, all healthcare personnel present during the planned sessions were invited to participate, regardless of their professional education or role. Participation was voluntary, and all individuals who expressed interest in the study were included; therefore, some groups had three participants, while others had seven. All participants had previous experience working in traditional nursing homes before transitioning to their roles in a DV, and most had experience working in dementia care. A complete presentation of the participants’ demographic data is presented in Table 1.

**Table 1:** Demographic data of the participants from both DVs

	Focus group number	Number of participants in each focus group	Gender	Age variation (years)	Work experience (years)	Title
DVA	1	7	6 females and 1 male	30–64	5–25	One nurse and six nursing aids
	2	5	5 females	45–63	16–40	One nurse, three nursing aids, and one care assistant
	3	5	3 females and 2 males	35–64	12–45	One nurse and four nursing aids
	4	5	4 females and 1 male	33–47	2–12	One nurse, three nursing aids, and one care assistant
	5	5	2 women and 3 males	30–63	15–33	Five nursing aids
DVB	1	5	3 females and 2 males	45–55	16–26	One nurse and four nursing aids
	2	3	2 females and 1 male	37–58	17–22	One nurse, one nursing aid, and one care assistant
	3	5	3 females and 2 males	34–47	7–20	One nurse, two nursing aids, and one care assistant
	4	6	5 females and 1 male	30–60	3–25	Two nurses, two social workers, and two nursing aids
	5	7	7 females	20–61	1–43	Two nurses and five nursing aids

### Data collection

Ten focus group interviews, with five conducted at each DV, were conducted over eight weeks from October to November 2024. Focus group interviews, chosen for their efficiency, enable the exploration of diverse perceptions, with participants building on one another’s statements, thereby generating new insights and information [45]. For this study, focus group interviews were considered appropriate as a data collection method because healthcare personnel worked within a shared organisational and professional context, and their experiences are often shaped through teamwork and collective practices. The focus group format facilitated interaction and discussion among participants, enabling them to reflect on and build on one another’s perspectives, thereby enriching the data and providing insight into everyday care activities, challenges, and shared understandings. Conducting the focus groups at the beginning or end of scheduled work shifts in a familiar setting further supported participants’ comfort and openness. The method was therefore well-suited to capturing both individual viewpoints and collective dynamics relevant to healthcare practices within DVs.

The interviews were digitally recorded using Diktafon, a smartphone application. The application securely encrypts the recordings and transfers them to Nettskjema, a secure storage platform that automatically transcribes the interviews. Each research pair, acting as moderator and co-moderator, was responsible for transferring the interview recordings to Nettskjema and subsequently reviewing the transcripts by listening to the audio recordings and reading the text. Minor revisions to specific words and sentences were made as necessary to avoid ambiguity and ensure accuracy.

The focus group interviews lasted between 49 and 61 min. All the researchers and authors of the manuscript were involved in data collection. The interviews were conducted by pairs of researchers, with one serving as the moderator and the other as the co-moderator. Eleven researchers, comprising ten females and one male, are employed at the Department of Nursing and Health Promotion at Oslo Metropolitan University. Two additional researchers, one male and one female, serve as quality managers in their respective DVs.

All researchers have experience with qualitative research and conducting focus group interviews. Each focus group was conducted similarly, with the moderator beginning by explaining the study’s aim and inviting participants to pose questions for clarification. After addressing any necessary clarifications, the moderator collected demographic information from each focus group member and then asked questions according to the interview guide. At times, the co-moderator also asked questions or requested clarification.

The focus group interviews were guided by a semi-structured interview protocol adapted from a previous study [40], with specific themes and questions modified to align with the current study’s aim. In addition, the interview guide was reviewed several times for clarity and relevance. It was adjusted following discussions within the research group to ensure that the methods were perceived as relevant, understandable, and appropriate for the participants.

The interview guide contained five themes, ensuring that participants addressed topics relevant to the study’s aim, including questions about their experiences providing care in DV facilities. It also facilitated a comparison of how their caregiving practices are influenced by the characteristics of these DV facilities compared with those of traditional nursing homes. The themes and questions posed during the focus group interviews are presented in Table 2.

**Table 2:** Interview guide

Theme	Questions
The impact of the physical environment of the DV on the healthcare personnel’s caregiving activities	1. Can you describe how the physical environment influences your daily work, your caregiving practices/activities, and interactions/collaboration with residents in DVs? (For example, lighting, flooring, furniture, noise, access to outdoor areas, private rooms, common areas, views, nature, plants, opportunities for retreat, privacy, and social interaction, etc.)
The impact of the physical environment of the DV on healthcare personnel’s cooperation with each other	1. Can you describe how the physical environment influences cooperation with colleagues? (Reminders: rest areas for staff/reporting rooms, opportunities for team collaboration, etc.) 2. Please provide some concrete examples of how tasks are affected by the physical environment of the DV (How and why?) 3. Can you describe how the physical environment at this workplace affects your job satisfaction/well-being?
Professional understanding and professional identity in the DV	1. What experiences do you have regarding your own professional identity when more traditional care and support tasks are slightly altered? a. Who works in the store, who prepares the food, how do you manage task shifts, and how do you understand, for example, nursing in such a setting? b. Do you feel that you have the necessary resources to: - Perform your job effectively? - Provide person-centred care to the unique individual?
Ethics in the DV	1. Can you describe how physical design leads to fewer or more ethical challenges you face in your daily work? Can you compare this with experiences from traditional nursing homes? 2. Can you describe how you work with ethical challenges, and what role ethical reflection and management play in your practice? (For example, understanding and using informal coercion or nudging, how you address ethical challenges, and engage in ethical reflection/management of moral issues?)
Improvement potential of the DV	1. Can you suggest some improvements to the physical environment that could be made in the DV if a renovation project is planned?

In addition to these main themes and questions from the interview guide, several probing questions, such as “Can you elaborate more on …?” or “Why do you think so?” were asked to stimulate the participants to provide detailed information about their experiences and perceptions, or to clarify the provided information. The interviews took place in a meeting room at the participants’ workplace, either at the beginning of their evening shift or at the end of their day shift, to minimise disturbance. Except for participants and researchers, no other persons were present in the room.

### Ethics approval and consent to participate

This project does not constitute medical or health research as defined in the Norwegian Health Research Act [46] and therefore did not require prior approval from a Regional Committee for Medical and Health Research Ethics. The planned processing of personal data was notified to SIKT, the Norwegian Agency for Shared Services in Education and Research (formerly NSD) via SIKT’s Data Protection Services (notification submitted on 10.06.2024) and approved on 26.06.2024 [Ref. No. 894773]. Approval to conduct the study was also obtained from the Nursing Home Agency (Oslo Municipality) and from the management of each DV. The first author (DL) sent an email requesting the use of a modified version of Lee et al.’s [40] interview guide, which had been obtained before the study commenced.

The study was conducted in accordance with the principles of good scientific and ethical practice, as outlined in the Declaration of Helsinki [47], including obtaining informed consent, considering potential consequences, and maintaining confidentiality. Prior to the commencement of the study, all the participants received detailed information regarding the study’s objectives, data collection procedures, potential risks and benefits, confidentiality measures, and their right to withdraw at any time without consequence. All participants were informed that their participation was voluntary and that they would not receive any economic or other benefits. Although the DVs’ quality managers recruited them, they were under no obligation to participate. They were also assured that if they chose to withdraw from the study at any time, there would be no negative consequences for their employment at the DVs. All participants provided written consent to participate before the data collection. None of them chose to withdraw from the interviews. All data are de-identified and stored on a password-protected computer. Only the researchers involved in the study have access to the files. In accordance with ethical requirements and as stipulated by SIKT, all interview recordings will be permanently deleted after the project period has ended and the study results have been published. Anonymised interview transcripts will be retained and may be used for secondary analysis.

### Data analysis

The interview transcripts totalled 172 A4 pages. For analysis and insight, all data were analysed by hand. Although the first author had primary responsibility for the data analysis, all researchers, co-authors of the present paper, were involved in reading and analysing the interviews and in providing feedback on the analysis.

To analyse the data, a qualitative inductive content analysis, as described by Kyngäs [48], was employed. This analysis method includes three steps: (i) preparation, (ii) organising, and (iii) reporting the findings. In the first step, *preparation*, the researchers independently listened to the audio recordings and carefully read the text, sentence by sentence, to identify sentences relevant to the study’s aim and classify each as an open code. This process of reviewing raw data and marking occurrences of open codes contributed to reducing the data.

In the next step, *organising*, the researchers met to discuss the open codes and, by comparing similarities and differences among the codes, determined which codes could be grouped and given names. Discrepancies among researchers were resolved through discussion until a consensus was reached. In the final step, *reporting the findings*, the researchers presented the results by describing the content of each category, supported by participant quotations. An example of the coding tree is presented in Table 3.

**Table 3:** An example of the analysis - from codes to categories

Codes	Sub-categories	Category
“Fresh start” “Modern facilities” “More and better space” “Better lighting” “Feels more like home” “Encourages independence” “More room for activities” “Green and large outdoor areas”	New place – new opportunities	Adaptation to a new care environment
“Lack of resources and short-staffed” “Digital issues with the technology” “Unfamiliar design” “Storage issues” “Missing a designated room for healthcare personnel” “More household tasks than professional tasks” “Disorientation for both residents and healthcare personnel” “Poor communication during the transition” “Still adjusting” “Being a firefighter”	Challenges with moving into a new build	
“Letting go of old routines” “Stuck in the old ways” “Difficult transition” “Need to adapt” “Clinging to the past and traditional care models” “Learning a new system” “New expectations” “Time to adjust” “Staff resistance” “Mixed feelings”	Changing the mindset - forgetting the past	
“Making it work anyway” “Going the extra mile” “Adapting creatively” “Doing more with less” “Finding solutions” “Workload imbalance, but still managing” “Team effort”	Making the impossible work	

In inductive content analysis, data are analysed continuously as they are collected. This helps identify when new data no longer contributes additional codes. After conducting 10 focus group interviews, the existing coding framework adequately explained the data, and no new theoretical insights emerged, indicating theoretical saturation [49]. Moreover, by including participants with varying experiences and perceptions, the likelihood of reaching saturation was enhanced without prematurely stopping data collection.

### Rigour of the study

Elo et al. [50] recommend that trustworthiness in qualitative research should be maintained at all stages of the research process. The most widely used criteria for evaluating qualitative content analysis are those developed by Lincoln and Guba [51], which include credibility, dependability, confirmability, and transferability. However, according to Elo et al. [50], a common feature of these criteria is that they aim to support trustworthiness by accurately reporting the content analysis process.

The main trustworthiness issues identified during the preparation phase concerned the data collection method, the sampling strategy, and the selection of an appropriate unit of analysis. To address these issues, the researchers provided a detailed justification for using focus group interviews. Considerations such as identifying the most suitable sampling method and selecting appropriate informants led to the use of purposive sampling, the most common approach in content analysis studies [50]. Dependability, defined as data stability over time and across conditions [51], was enhanced by clearly describing inclusion criteria and participant characteristics, enabling assessment of transferability. Selecting an appropriate unit of analysis was also crucial for credibility; this was achieved through collaborative discussions among researchers until consensus was reached. The trustworthiness of the analysis was further supported by the inclusion of an example of the coding tree (Table 3).

In the organisational phase, trustworthiness focused on the clarity of categories, the level of interpretation, and the verification of findings. Credibility was ensured through member checking, whereby the first author (DL), together with AKB and CT, regularly met with selected participants from both DVs to discuss the ongoing analysis. Participants confirmed that the findings accurately reflected their experiences. Confirmability was strengthened by ensuring that interpretations were grounded in the data rather than the researcher’s assumptions. Following Elo et al. [50], the first author conducted the analysis, with the co-authors closely monitoring the process and all authors discussing and resolving any disagreements. Transparent explanation of category development further supported credibility.

Trustworthiness in the reporting phase was closely linked to the transparent presentation of results, methods, and analyses, particularly with respect to transferability, confirmability, and credibility [50]. Findings were reported systematically, with clear links between data and interpretations. Transferability was supported by presenting findings in a way that allows readers to judge their applicability to similar dementia care contexts. Confirmability, understood as objective representation of participants’ accounts [51], was ensured by faithful interpretation of the data and acknowledgement of the researcher’s positionality. The use of quotations further strengthened trustworthiness; selected quotes were jointly discussed, translated into English, and verified against the Norwegian originals to preserve meaning. Finally, the researchers enhanced trustworthiness by providing a clear description of the three phases of analysis and the relationship between the findings and the original data, thereby allowing readers to assess the dependability. A transparent decision trail enables other researchers to follow the analytical process, indicating the study’s high reliability.

## Results

The researchers’ interpretation of the data, based on their analysis and discussion, indicated that healthcare personnel perceived their experiences working in DVs as opportunities for growth, innovation, and adaptation to a new environment, highlighting positive outcomes and a future-oriented mindset. This overall perception is supported by three categories: (i) Adaptation in a new care environment; (ii) Managing residents’ increased freedom of movement; and (iii) Development of the caregiver role within DVs, which collectively reflect a shift in dementia care from traditional models to a more flexible and innovative approach, where healthcare personnel adapt their caregiving roles to meet residents’ individual needs through creative and person-centred solutions.

In the following section, the findings are presented with quotations from participants’ statements. Each statement is followed by a number indicating the focus group (FG) and from which DV the focus group was conducted (i.e., participant P in FG2 from DVA).

### Adaptation to a new care environment

This category explores the participants’ experiences transitioning from traditional nursing homes to DVs, a process described as both rewarding and challenging. The shift to a novel care model brought moments of joy but also uncertainty as healthcare personnel adjusted to new routines, unfamiliar layouts, and a departure from established practices. Although initially feeling unprepared, participants were committed to adapting and building meaningful relationships with residents in this new care setting.

Healthcare personnel described the transition as entering a world where traditional workday structures and routines no longer applied. They were on the frontlines of “making the impossible work,” striving to implement a care model to enhance the autonomy and dignity of residents. Although the vision was inspiring, it required resilience, creativity, and determination to manage the complex transition from traditional practices to DV ideals. One participant said:

One positive aspect, of course, is the new facility. The modern facility offers greater comfort for the residents and us. However, it’s not ideal, yet… So, when we arrived, we were told, ‘This is how it will be,’ and we had to adapt and learn from there (P-FG4-DVA)

In the early stages, the unfamiliar infrastructure and unpredictability of daily routines created a sense of disorientation. Participants recalled feeling overwhelmed by the layout and the demands of working in unfamiliar physical environments with newly transferred residents from traditional nursing homes. Over time, however, this initial stress began to subside, as one of the participants revealed:

I think it was much more difficult at the beginning when we first moved here. We moved during the spring and were among the first to arrive. It was very hectic and challenging to navigate. As nurses, we were assigned to different units and had to get to know new residents. It was a lot to handle and quite stressful. But now, as I see it, things are starting to settle, and I feel like I’m beginning to get familiar with everything … (P-FG2-DVB)

Interestingly, some participants reported that although they struggled to adjust to the new work environment, residents, particularly those from traditional nursing homes, adapted more quickly than expected. Despite their familiarity with locked doors and long corridors within traditional nursing homes, the residents gradually came to appreciate the freedom and safety of the new setting. One of the participants explained:

I have transferred from a nursing home to DV. Most residents were used to the long corridors and locked doors of the previous facility. Witnessing their adjustment to the new environment was striking. At first, they were very cautious, hesitantly asking, ‘Can I go out here? Is it okay?’ It took some time, but they eventually started to feel safe and comfortable in their new surroundings … (P-FG1-DVA)

One significant challenge highlighted by participants was the DV’s physical design. Although intended to replicate a village-like atmosphere and promote a sense of normality, the complex layouts often created confusion. Healthcare personnel described navigating the building as akin to a labyrinth, with poorly marked pathways and disorienting architecture that complicated daily routines for both healthcare personnel and residents. One of the participants stated:

I can say that this building is unique, at least. It’s somewhat complex in terms of its structure. And that was what shocked us the first time … as staff, it took us some time to get accustomed to the pathways and routes within and between the units, from one place to another. Some residents still struggle to find their way back after being outside in the garden. They get lost and end up in other areas. It takes considerable time to find them. (P-FG3-DVB)

The confusion was not limited to outdoor areas. Indoors, the absence or intentional omission of signs made navigation equally difficult. One participant reflected on the learning curve and the puzzling design choices and shared her experience:

You also see it when you go indoors. When I was new here, I felt like I was in a maze and couldn’t find my way. I also found the signs a bit challenging. Over time, I figured out that if I go in here, I’ll also end up on … [name of the unit], but there’s no sign indicating that. I asked why there wasn’t a sign, and the explanation was that there was no desire to guide residents through that door … (P-FG4-DVB)

Despite these physical challenges, most participants viewed the DV concept with optimism. They recognised the new environment as an opportunity, one that allowed for the redefining of relationships between staff and residents and for providing care in a space truly designed for people with dementia. Architecture became more than a structure; it symbolised potential and purpose. One of the participants explained:

I applied here because I wanted to work with people with dementia in an environment designed for them. I didn’t know much about the concept itself, but I knew it was a ‘concept’ [laughter] … So, what I thought here was that working in an environment where things are, in a way, set up for them or are meant to be set up for them, was important. And then there’s something about using the environment you work in in a meaningful way so that you make the most of the concept … (P-FG3-DVB)

### Managing residents’ increased freedom of movement

The participants described the DVs as a care concept that offers residents significantly more freedom compared to the restrictive environments of traditional nursing homes. This increased autonomy was seen as beneficial, enhancing residents’ dignity and sense of independence. However, this freedom also introduced new challenges, particularly concerning safety. Healthcare personnel often felt conflicted; on the one hand, they wanted to encourage residents to go outside and enjoy the environment; on the other hand, they had to protect them from wandering into potentially unsafe areas or engaging in risky behaviour. Despite these concerns, the staff worked hard to find a balance between empowering residents and ensuring their well-being. One of the participants said:

It’s a bit of both… On the one hand, they benefit from being able to go outside. On the other hand, they may not know where they’re going or what they want to do; however, simply having the opportunity to leave the building, wander around, and do things on their own makes a significant difference for them. (P-FG1-DVA)

All participants emphasised the importance of freedom of movement. Unlike traditional nursing homes, where residents must adapt to institutional rules and routines, DVs were designed around residents’ needs, reducing confinement and allowing them to be more themselves. This was perceived as contributing positively to the residents’ quality of life. One of the participants shared a positive experience:

They have a walking group as a weekly activity. I was shocked to see so many of them going out in the rain. I wouldn’t have bothered … [laughter]. But the residents were very eager to participate. They’ve been asking about it every Tuesday when they go walking with poles. They take long walks and then come back, and they do it regardless of the weather, whether it’s rain, sunshine, or snow … (P-FG2-DVB)

However, this freedom is limited to within the DV’s boundaries. According to participants, each resident was assigned a key bracelet, which allowed them to move around the DVs’ area. This limitation raised ethical dilemmas about how to balance autonomy with safety. One of the participants expressed her disappointment at being unable to provide a greater sense of freedom to one of the residents:

I have a patient in … [name of the unit]. Although the concept is that residents can move freely, I observe that as soon as she reaches the reception area and finds the door locked, she becomes upset. She comes back, saying, ‘This is a prison.’ We try to explain, ‘But you can go wherever you like,’ yet she responds, ‘But I can’t get out.’ While she is free to move around within the DV, she feels restricted because she can’t go beyond its fences … (P-FG3-DVB)

After transitioning to the DVs, healthcare personnel often described their roles as resembling ‘firefighting’. They found themselves constantly responding to urgent situations, from managing residents’ behavioural outbursts to addressing logistical emergencies, such as fixing meals, cleaning, or resolving technological malfunctions. Most participants agreed that this reactive approach left little room for proactive care planning or for addressing residents’ psychosocial needs. They all felt drained by the continuous pressure and lack of time to reflect and plan, as expressed in the following statement:

We discuss situations with each other if they arise, but we don’t have time to ask, ‘What can we do for her to improve her daily life, right?’ We don’t have time to focus on a resident unless something specific happens … (P-FG4-DVB)

On the other hand, the same participant, despite challenges and time pressures faced daily, expressed a profound sense of dedication, compassion, and commitment to caregiving:

The residents – I love my residents. I am passionate about them. I would go through fire and risk my life for them. They are my priority, and I want them to thrive and feel well … (P-FG4-DVB)

The integration of care technology in DVs received mixed feedback from participants. Most of the participants were technologically competent and appreciated how these tools, including electronic patient journals, location-tracking technology-enabled wristbands, digital monitoring systems, and DVs’ communication platforms, helped streamline administrative tasks and enhance resident safety. One of the participants conveyed her optimism as follows:

… At the same time, I have care technology that makes me feel secure and allows me to keep an overview of what is happening inside … [name of the unit] (P-FG1-DVA)

Another expressed their enthusiasm as follows:

So, it’s great [the care technology]. They [the residents] get to be themselves, and many of the residents have location-tracking technology in their key bracelets; therefore, they can go in and out freely, and they are being monitored (P-FG2-DVB)

A third participant helpfully explained how care technology works:

All the residents wear an alarm necklace. We use an application. We must log in to the application upon arrival at work. For example, I can see where the residents are located. And we receive alerts when they end up in a place they shouldn’t be, such as on the stairs or elsewhere … [assessed as a dangerous place]. So, my experience with care technology is fantastic! (P-FG3-DVB)

At the same time, several participants noted that more training was needed. Some care technology systems did not always function as intended, and residents were often unable to operate the technology independently. For example, the light switches were so modern that the residents struggled to figure out how to use them and required assistance. One of the participants explained:

It was so technical that we had to undergo training to learn how to do it, and the residents were unable to manage it. They can’t do it. So, we constantly must help with turning the lights on and off in the room … (P-FG2-DVB)

Another critical issue that emerged during the focus groups was the composition of the resident groups. Participants emphasised that to benefit from the DV environment, residents need a certain level of physical and cognitive function, despite a dementia diagnosis. Many residents, however, were unable to engage in the activities or navigate the space due to impairments, as one of the participants said:

They end up not being able to get out of the elevator, and then they’re terrified … and they use it [the elevator] as a toilet, in there, in the dark. This has happened many times… (P-FG4-DVA)

To ensure a more positive experience, participants emphasized the importance of grouping residents by ability level. One participant described how they made a successful reorganisation of a resident group:

Firstly, we need to have similar residents in the group … In the beginning, we had a mix of psychiatry, Alzheimer’s, vascular dementia, alcohol abuse, and a high number of incontinent patients altogether. We had to sort this out. Meanwhile, the most well-functioning residents, four women who weren’t too old, ended up together with two quiet men, and that became a great group … (P-FG3-DVA)

### Developing the caregiver role within DVs

All participants shared a common experience: every aspect of the DVs, from their layout to their furnishings, was thoughtfully designed to improve residents’ quality of life by providing comfort and security. They agreed that the environment itself actively supports care by encouraging meaningful activities and promoting independence. However, as several participants pointed out, the DV is also their workplace, and the design must balance residents’ well-being with the practical needs of healthcare personnel to ensure the provision of effective and efficient care.

Most of the participants agreed that entering the DV care environment required a shift in mindset. Participants emphasised that this transition didn’t mean discarding experience but rather letting go of routinised care to embrace new ways of thinking. They all agree that the philosophy of care in DVs calls for adapting the environment and routines to the residents, not the other way around. According to participants, this shift demanded both courage and vulnerability, as they had to trust their intuition and creativity in the absence of a straightforward guidebook. This transformation was captured in a dialogue between two participants from FG3, DVB:

P1: Because right now, it’s not just a new concept but also a new way of working. That’s how I see it. You’re not supposed to work the same way [as they did in traditional nursing homes].

P3: To use the environment correctly, you also have to establish a way of working so that the environment works with you.

Participants also noted that residents from both DVs were transferred from different traditional nursing homes, thereby introducing additional challenges. They had varying degrees of cognitive impairment, along with diverse physical health conditions and high-demand care needs. However, when the DVs were designed, the staffing levels were initially calculated based on the assumption that residents would be largely independent in managing daily activities. In practice, residents required significantly more assistance, particularly with tasks like cooking and cleaning. As a result, the role of healthcare personnel expanded, resulting in increased workloads and responsibilities not accounted for in the original staffing model. One participant described the situation:

At times, depending on the day of the week, we are alone in a unit with six residents. We can have residents with psychiatric symptoms, entirely care-dependent residents, bedridden, or in wheelchairs … everybody placed in the same unit, and you are alone. You must prepare breakfast and lunch, which entails physically going to the store to purchase groceries. Initially, we even had to prepare dinner from scratch. We must wash clothes, fold them, clean and tidy the rooms, and dust. Everything in every room … (P-FG3-DVA)

Although other employees performed the most demanding cleaning tasks, most participants reported taking on numerous additional duties, including cooking, cleaning, and laundry. While these domestic tasks were initially intended to be carried out in collaboration with residents, residents’ high level of functional impairment often necessitated that healthcare personnel manage these tasks independently. This left less time for direct caregiving. As one participant explained:

We prepare breakfast, homemade lunch, hot lunch, dinner, and evening snacks. Many new staff members are shocked and feel like kitchen help because they have little time for anything else. After breakfast, they need to clean, shop, and restock their supplies. Then, they quickly prepare lunch, serve it, and attend to laundry and medication. This leaves less time together with residents (P-FG1-DVA)

All participants expressed a strong desire to provide high-quality care. With the prioritisation of domestic tasks, some felt that their professional skills were underutilised. One participant shared her experience:

I feel like we are regressing professionally. I’m forgetting much of the professional aspects of being a healthcare worker. For us, nursing aids, the focus has shifted more towards being a homemaker, kitchen assistant, cleaner, or janitor – whatever you want to call it. I feel that the professional side is completely gone. However, we are expected to conduct clinical observations and provide feedback. Unfortunately, there isn’t enough time for that, is there? (P-FG3-DVA)

Despite these concerns, other participants appreciated the flexibility and independence the DV model offered, as seen in the dialogue below between two participants from FG5 – DVA:

P2: There isn’t the same pressure here. No one comes to me afterwards asking, ‘Why hasn’t he [the resident] showered?’ It’s a more independent way of doing things. I enjoy working here.

P4: When we moved here, I truly felt like I contributed a lot as a healthcare worker. I feel in control. When I go home, I am satisfied because I manage most things on my own. You think that you’ve contributed, and when you go home, you know you’ve made a difference.

Although much of the physical design of the DVs was perceived as positive, a drawback was that not all units in the DVs had a designated room for healthcare personnel to provide reports or to take breaks. This concern was related to how the architecture of the DV units influenced healthcare personnel’s activities. Since each unit was designed to have a homelike feel and perception, no room was designated for healthcare personnel to use as a report room, to document observations, to conduct discussions and consultations with colleagues, or to plan care activities. One of the participants stated:

We don’t have a staff room where we can sit and document and write our reports. We must do it while sitting with the residents in the common area. And I think it works quite poorly. You get interrupted… There’s always someone coming up to ask you something. Whether it’s a resident or someone visiting, that is a bit of an issue … (P-FG3-DVA)

## Discussion

This study aimed to explore healthcare personnel’s experiences transitioning from traditional nursing homes to the dementia village care model in Norway. Three categories emerged from the data analysis, mirroring healthcare personnel’s experiences working in DVs as opportunities for growth, innovation, and adaptation to a new environment. These three categories are: (i) Adaptation to a new care environment; (ii) Managing residents’ increased freedom of movement; and (iii) Development of the caregiver role within DVs. The transition from traditional nursing homes to DVs represents a cultural and structural shift in dementia care. Participants’ experiences reflect both the promise and the complexities of implementing such a different care philosophy, one that emphasises autonomy, dignity, and normalised living environments for people with dementia.

### The challenges and opportunities of the transition

The participants’ experiences reflect the broader discourse on person-centred care, where physical environments are not merely backdrops but active agents in supporting residents’ autonomy and well-being [52].

DVs embody this philosophy through architecture that reflects ordinary community life, aiming to deinstitutionalise, transform, and normalise care for people with dementia [32]. However, the findings revealed that while residents appeared to acclimate relatively quickly, healthcare personnel experienced high levels of stress in the early stages of the transition, a typical response to complex environmental and procedural changes. These findings are similar to those reported by Brouwers et al. [53], who described how healthcare personnel experienced feelings of being lost, both physically and emotionally, when they relocated from a traditional nursing home to an innovative living arrangement.

Although healthcare personnel experienced high levels of stress during the relocation, they were still required to continue providing care as part of their daily routine, findings similar to those from another study [54]. This added pressure increased their perceived stress, as they were expected to offer emotional support to residents and maintain a positive attitude despite their psychological strain. This dynamic can lead to ‘emotional dissonance’, the conflict between felt emotions and those that must be expressed professionally, as noted by Fiabane et al. [55]. This conflict has been linked to emotional exhaustion and reduced job satisfaction among healthcare personnel.

However, relocation is a continuous process that begins with preparation, extends through the move, and continues into the post-relocation phase, which can last several months [56]. Although the interviews were conducted during the post-relocation phase at both DVs, participants retained vivid memories of the relocation phase itself, which revealed experiences that negatively affected their caregiving activities for residents and the challenges they faced. Similarly, results from earlier studies indicate that relocation can affect healthcare personnel’s social relationships and lead to increased isolation and uncertainty [56].

Experienced stress can, in turn, lead to increased absenteeism [57], which can be detrimental to healthcare institutions amid existing staff shortages. Moreover, changes in organisations are not always readily accepted because they conflict with fundamental human needs for a sustainable environment [58]. Results from an integrative review [59] investigating the reasons for resistance to change in nursing indicate that individual, interpersonal, and organisational factors may contribute to reluctance to change.

The transition from a traditional nursing home with familiar routines to a new and, at times, challenging physical environment of the DV, while remaining committed to delivering high-quality care that met residents’ diverse healthcare needs, was often difficult for participants to accept. During the relocation phase, these changes were perceived as negatively affecting healthcare personnel’s willingness to adapt at the individual level. Although the challenges of the relocation phase were described in detail in the interviews, some participants in the current study reported satisfaction and positive experiences, indicating variation in adaptation among healthcare personnel. This disparity in adaptation aligns with the findings of Broekharst et al. [60], who examined pre-relocation, bridging, and post-relocation initiatives used for relocations within and between nursing homes. Their study revealed that relocations commonly induced stress, anxiety, burnout, and additional workload for healthcare personnel. Similarly, the participants in the current study experienced uneven adaptation, with some individuals struggling more than others during the relocation process. The initial feeling of ‘making the impossible work’, expressed by some participants, reflects some of the challenges reported in a study exploring employees’ reactions in organisational change processes [61]. The participants faced ambiguity due to unclear protocols and shifting job expectations, resulting in ‘transition pain’, an emotional response to organisational change triggered by disruptive external factors [61]. Despite transitional challenges, similar to findings from another study [38], all participants emphasised their commitment to providing dignified care to residents, thereby enabling a familiar and meaningful everyday life in the DV.

### Balancing residents’ autonomy and safety

The experiences of healthcare personnel working in DVs reflect a complex interplay between promoting resident autonomy, ensuring safety, managing digital tools, and navigating ethical boundaries. While participants generally viewed this shift as positive for residents, they also encountered substantial challenges, particularly in balancing freedom and risk, managing new digital solutions, and supporting diverse resident needs.

A notable experience reported by participants following the establishment of the DVs was an increased sense of resident autonomy. Unlike traditional nursing homes, DVs enable residents to move more freely within the physical environment, thereby providing access to outdoor spaces and community-like settings. This was made possible by the integration of care technology, including digital monitoring systems such as location-tracking technology, which is used in wristbands and alarm necklaces. Participants described how this ability to walk outdoors, even without a specific destination, significantly improved residents’ well-being and sense of normality. These findings align with a study conducted by Goodall et al. [62], which emphasises that care technology-supported environments and thoughtfully designed spaces can enable meaningful, personalised activities and enhance person-centred care for residents. One of them, SENSE-GARDEN, is a digital innovation that combines multisensory stimuli with digital media to create personalised environments for people with dementia and residents of DVs [62]. However, the integration of care technologies was met with both optimism and criticism. On the one hand, participants acknowledged that these tools streamlined workflows and enhanced safety, particularly with location-tracking technology-enabled devices that enabled more unrestricted resident movement. On the other hand, concerns have been raised about the functionality, reliability, and user-friendliness of these systems, particularly for the residents of the DVs. In their study, Iancu and Iancu [63] reinforced the idea that design principles and solutions should align with the physical and cognitive profiles of older adults, thereby contributing to the development and use of appropriate devices for this population by researchers and clinicians. The participants in our study called for more comprehensive training to ensure that technology enhances rather than hinders the provision of care. According to Golz et al. [64], digital competence can help prevent technostress and enable healthcare personnel to manage expected disruptive changes. The participants reported challenges associated with the use of digital devices, which can increase healthcare personnel’s stress levels and negatively impact the quality of care they provide. These findings align with the results of a recently published cross-sectional study conducted by Ylönen et al. [65], which emphasises the importance of digitalisation, particularly AI-driven technologies in social services and healthcare. However, according to Lerzynski [66], neither technology alone nor existing legislation can fully address the numerous ethical concerns. One of them, as revealed in the present study, shows that increased autonomy introduces ethical dilemmas and practical tensions. The participants often found themselves torn between encouraging freedom and ensuring safety, especially when residents were prone to wandering or engaging in potentially risky behaviour, such as being locked in an elevator for many hours. Although location-tracking bracelets and boundary-limited access systems offered some peace of mind, they also symbolised a limitation of true freedom. As one participant reflected, a resident described the locked exit as turning the village into ‘a prison’, a comparison used also in an early study conducted by Heggestad et al. [67]. This emotional contradiction highlights the ethical tension between promoting autonomy and restricting movement for the sake of safety.

Participants also raised concerns about their daily working conditions in the DV model, often describing their roles as ‘firefighting’, constantly reacting to incidents, residents’ behaviour issues, and digital malfunctions. This reactivity left little time for reflection, care planning, or relationship-building. This illustrates how new care models, such as DVs, can strain healthcare personnel’s capacity when systems and support mechanisms are not concurrently adjusted. Similarly, results from a systematic review and meta-analysis conducted by Costello et al. [68] demonstrated that healthcare personnel caring for people with dementia frequently experience moderate emotional exhaustion, particularly when faced with challenging residents’ behaviour, insufficient staffing, poor leadership, and inadequate organisational support. The ideal of person-centred care may fall short when healthcare personnel lack time or training, or feel emotionally exhausted, making it difficult for them to deliver it effectively. Despite these pressures, healthcare personnel consistently demonstrated profound dedication and emotional commitment to the residents. One participant described being willing to ‘go through fire’ for the residents they cared for. This commitment was also reported in a study by Midtbust et al. [69], which found that healthcare personnel relied on their moral resilience to continue providing compassionate care, and that the healthcare personnel navigated emotionally charged situations with empathy, maintaining care standards aligned with their ethical values despite the ongoing pressures of their roles.

Another recurring issue involved the heterogeneity of resident cognitive functioning and physical abilities. Participants emphasised that the DV model is most effective when residents have comparable levels of physical and mental function. In cases where residents were too impaired to navigate the environment or engage in group activities, healthcare personnel observed distressing incidents, such as residents misusing elevators due to disorientation. A well-balanced group composition is crucial for ensuring that all residents benefit from the DV’s open and autonomy-supportive environment. Quirke et al. [70] emphasised that environmental design, including space layout, circulation patterns, and activity zones, must be paired with tailored strategies for residents’ placement to foster both resident safety and social inclusion. Their study highlights that thoughtfully designed environments can significantly support cognitive accessibility, well-being, and independence when residents are grouped by similar functional ability. Without such intentional alignment between design and resident capabilities, even the most well-conceived environments may fail to deliver their potential benefits [70].

### Embracing the new caregiver role

The development of the caregiver role in DVs represents a profound transformation from the institutionalised routines of traditional nursing homes to a more person-centred, holistic, and innovative approach. This transformation is deeply rooted in environmental design and the reconfiguration of staff roles.

All participants highlighted that the physical and social environments in DVs are intentionally designed to promote residents’ autonomy, familiarity, and engagement. In their study, Quirke et al. [70] emphasised that environmental design is increasingly recognised as a valuable non-pharmacological approach to managing dementia symptoms, thereby contributing to greater independence and well-being among individuals living with various age-related cognitive, physical, and sensory impairments. The home-like design not only supports the residents’ orientation but also reduces agitation and increases participation in activities [52]. However, healthcare personnel must learn to navigate this dual-purpose environment: a residence for residents and a workplace for them. This balance echoes the biopsychosocial model of dementia care [10], which emphasises integrating physical, emotional, and social aspects into the caregiving context [13, 31]. A critical insight from some participants was the need to ‘let go’ of traditional, task-oriented routines in favour of more flexible, resident-centred practices that could be adapted to the individual needs, pace, and preferences of residents. This aligns with person-centred care principles [71, 72], in which routines are adapted to meet residents’ needs rather than imposed upon them. Staff were encouraged to use their intuition and creativity, qualities often underemphasised in routine-based institutional care. Such a shift involves not only a redefinition of professional identity but also emotional vulnerability. Similar to results from a previous study [73], the current study demonstrates that the absence of clear protocols compels healthcare personnel to engage in improvisational caregiving, in which relationship-building, emotional intelligence, and contextual judgment are critical.

Despite the ideal of collaborative living, many participants reported increased workloads due to residents’ higher-than-anticipated needs. The original DV model assumed a resident population capable of participating in daily activities, such as cooking and doing laundry. However, many residents were unable to do so due to significant cognitive or physical impairments. This situation mirrors findings from a study by Zwakhalen et al. [74] on small-scale, homelike dementia care settings, which found that staffing models were based on assumptions of greater resident independence than was actually present. In reality, healthcare personnel often assumed integrated domestic roles, including personal care, medical duties, household tasks, and organising activities, to compensate for residents’ limitations. These additional responsibilities were associated with an increased workload and a risk of role strain, thereby reducing the time available for clinical care and observation.

In the present study, several participants perceived that their knowledge and clinical skills were compromised by the domestic nature of the role they had recently assumed. The combination of caregiving with domestic chores reflects the ‘blurring of boundaries’ [75] in caregiving roles in DVs and similar environments. While the domestic setting supports a therapeutic milieu, it can unintentionally deskill healthcare personnel if not carefully managed or supported by proper role delineation, particularly when only one person is on duty.

Despite these challenges, other participants found the DV model empowering, appreciating the autonomy, trust, and flexibility the model afforded. These findings align with research suggesting that greater autonomy in care roles correlates with higher job satisfaction and lower burnout [76]. Staff who felt they could make meaningful decisions and see the direct impact of their care reported increased fulfilment.

Participants consistently raised concerns about the lack of a private, dedicated space for healthcare personnel, which limited their ability to document care, engage in professional discussions, and take breaks. In striving to maintain a non-institutional, homelike atmosphere, DVs may inadvertently overlook the operational and psychological needs of healthcare personnel. This concern is supported by the results of a systematic review by Shetty et al. [77], who emphasise that the physical environment in healthcare facilities significantly affects staff performance, stress levels, and overall well-being. The review highlights that insufficient access to private or semi-private areas for healthcare personnel to document, discuss subject-specific or clinical issues, reflect, and rest can lead to increased cognitive fatigue, reduced job satisfaction, and ultimately, a decline in the quality of care. The review reinforces the importance of incorporating well-designed, staff-specific spaces within care environments, even those designed to feel like home, to ensure a balance between resident-centred care and the professional needs of those delivering it. Furthermore, the results from the review [77] support participants’ descriptions from the DV setting, in which maintaining a homelike atmosphere unintentionally led to the omission of essential staff spaces. This design trade-off risks impairing both the quality of care and the performance of healthcare personnel.

Ultimately, the findings highlight the challenges of delivering care while maintaining professional expertise. The DV model challenges healthcare personnel’s thinking about traditional care provision, demanding creativity, flexibility, and resilience. Some healthcare personnel thrive in a free environment, while others feel lost without structure. Still, what emerges is a new kind of professional role: one who can cook a stew while recognising early signs of delirium, who comforts a resident during sundowning while folding residents’ clothes just the way they like. This multifaceted and partly new role is both demanding and, for many participants, deeply rewarding.

### Implications for clinical practice

The findings from this study suggest several important implications for clinical practice in DVs. First, there is a need to reconceptualise the role of healthcare personnel to reflect their hybrid and multifaceted nature, one that blends clinical, domestic, and relational responsibilities. Healthcare personnel are no longer solely clinical practitioners but also facilitators of everyday life, a role that requires education and clinical skills that extend beyond traditional caring competencies to include relational care, environmental engagement, and creative problem-solving.

Second, staffing models in DVs must be reassessed to align with residents’ actual cognitive and physical functioning, as many require far more assistance than initially anticipated. Without adequate staffing, the increased burden of domestic chores risks overshadowing essential caring duties, leading to job dissatisfaction and a decline in professional skills.

Third, clinical environments should ensure that healthcare personnel have access to dedicated, private spaces for documentation, discussions, reflection, and collaboration, spaces that support the professional side of care without compromising the homelike atmosphere of the DV. Additionally, emotional and professional support structures must be strengthened through reflective supervision, mentoring, and opportunities for staff to participate in shaping routines and care strategies. Moreover, care technology used in DVs must operate reliably to ensure both effective care delivery and resident safety. Healthcare personnel should receive appropriate education to develop the digital competencies and skills necessary to integrate these tools into their daily practice effectively.

Ultimately, it is essential to safeguard and reaffirm the professional identity of healthcare personnel by offering ongoing clinical training and ensuring that caregiving remains complex and nuanced within this reimagined DV model of care.

### Strengths and limitations of the study

This study provides valuable insights into the evolving role of healthcare personnel in DVs; however, several limitations should be acknowledged. First, the findings are based on a qualitative dataset drawn from ten focus groups in two specific Norwegian DV settings, which may limit the transferability of the results to other care environments or cultural contexts. Additionally, the participant sample consisted solely of healthcare personnel who had already adapted to the DV model, potentially introducing positive response bias or underrepresenting more critical or resistant perspectives. The absence of longitudinal data also limits the ability to assess participants’ experiences and the evolution of their roles over time. The absence of residents’ perspectives may also be a limitation, as their lived experiences of living in a DV and being care recipients may differ from those of healthcare personnel who provide care. However, the study has notable strengths. To the best of our knowledge, this is the first study to explore healthcare personnel’s experiences working in a DV. It captures rich, first-hand experiences from diverse healthcare professionals, offering depth and nuance that quantitative methods might overlook. The use of direct quotations adds authenticity and credibility to the findings. At the same time, the focus on the intersection between environment, the provision of person-centred care, and professional identity addresses a gap in existing research. Furthermore, the inclusion of reflective dialogue among participants in the focus groups strengthens the inductive content analysis, thus highlighting the complex, lived realities of caregiving in DVs as innovative settings for dementia care.

## Conclusions

This study offers a nuanced understanding of how the role of healthcare personnel as caregivers is evolving within DVs, highlighting both the opportunities and tensions that emerge when more flexible, care technology, and person-centred approaches replace traditional models of care. The findings revealed that while the thoughtfully designed environments of DVs are perceived as supportive of resident autonomy, well-being, and meaningful activities, they also introduced significant demands on healthcare personnel. Participants described a need for a shift in mindset in which conventional care strategies were replaced by intuitive, relational, and creative forms of caregiving, in the absence of clear guidelines or formal structures. Although many participants appreciated the work autonomy in DVs, some voiced concerns about the increasing workloads, underutilisation of their clinical skills, and prioritisation of domestic chores such as cooking, laundry, and cleaning.

The lack of designated spaces for documentation and professional collaboration further complicated their ability to fulfil resident clinical responsibilities. These findings suggest that while the DV as a care model in dementia succeeds in rehumanising care for residents, it also risks professional dilution if organisational support is absent. To address this, clinical practice must reconceptualise caregiving roles and provide training that integrates the culture of DV as a model of care for people with dementia. A professional identity must be preserved through structured opportunities for skill development and leadership that validate the emotional and practical complexities of working in this innovative care model. In doing so, DVs can fully realise their vision, not only as environments that dignify ageing with dementia, but also as supportive workplaces for healthcare personnel. These settings should empower healthcare personnel to provide dignified care and to thrive within a collaborative culture that helps prevent work burden and reduce job-related stress.

## Electronic Supplementary Material

Below is the link to the electronic supplementary material.

Supplementary Material 1

## Data Availability

The datasets generated and/or analysed during the current study are not publicly available due to participant confidentiality agreements but are available from the corresponding author on reasonable request.

## Footnotes

1 The founders of De Hogeweyk, the first dementia village, recommend avoiding the term ‘dementia village’ and instead refer to it as the De Hogeweyk Care Concept. In Norway, there is a prevailing preference for the term ‘garden city’ [hageby]. However, due to the widespread international recognition of the term ‘dementia villages,’ this terminology is being adopted here.

## Abbreviations

DV: Dementia Village
DVA: Dementia Village A
DVB: Dementia Village B
P: Participant in focus group
FG [nr.]: Focus group [nr
